# Lowe Syndrome and Me: a co-creation video series connecting patients, caregivers, and researchers

**DOI:** 10.3389/fcell.2025.1610207

**Published:** 2025-06-05

**Authors:** Theresa Haugen, Jennifer L. Gallop, Hélène Doerflinger

**Affiliations:** ^1^ The Center for the Advancement of Art Education, Osage, IA, United States; ^2^ Lowe Syndrome Association, Cincinnati, OH, United States; ^3^ Gurdon Institute, University of Cambridge, Cambridge, United Kingdom; ^4^ Department of Biochemistry, University of Cambridge, Cambridge, United Kingdom; ^5^ Department of Genetics, University of Cambridge, Cambridge, United Kingdom

**Keywords:** co-creation, patient involvement, actin, phosphatidylinositol (4, 5)-bisphosphate, Lowe syndrome, oculocerebrorenal syndrome

## Abstract

Lowe syndrome (LS) is a rare genetic disorder leading to significant physical and cognitive impairments. Recognizing the need to bridge the gap between researchers and the LS community, a collaborative patient and public involvement (PPI) project, Lowe Syndrome and Me, was initiated. This initiative aimed to foster understanding, improve communication, and strengthen advocacy through a co-created video series. Researchers from the Gurdon Institute (United Kingdom) and caregivers from the Lowe Syndrome Association (United States) collaborated to develop a series of videos capturing the unique perspectives of patients and their families, advocacy group members, and researchers. Participants received video production and scriptwriting training, ensuring authentic representation and shared ownership of the content. The videos were disseminated through social media, research institute and patient group websites in the United States, United Kingdom and France, raising awareness and improving engagement within the LS community. Feedback from participants highlighted high satisfaction, increased understanding of research, and enhanced communication skills. Challenges included geographical barriers and limited participant diversity, but the project successfully fostered reciprocal learning and strengthened advocacy networks. This case illustrates how meaningful PPI can empower patient communities, enhance research relevance, and promote broader public awareness of rare diseases.

## Introduction

Lowe syndrome (LS), also known as oculo-cerebro-renal (OCRL) syndrome, is a rare genetic condition caused by mutations of the *OCRL1* gene (Matteis et al., 2017). It affects approximately 1 in 500,000 people, most of whom are males. The condition causes physical and cognitive disabilities and medical problems stemming from the three major organ systems involved (eyes, brain, and kidney). While researchers are in labs conducting cellular research, searching for paths that will positively affect the lives of those living with LS, families affected by LS are busy with daily medical needs, therapies, and care. Within their paradigms, researchers and affected families often have only a distant awareness of the other. Patient groups may not fully understand how research is conducted or how results at the cellular level could impact their lives. Researchers may have only theoretical clues about how the condition affects patients. Patient advocacy groups must understand family and patient needs as well as how research is conducted to inform patient groups and donors and allocate research funding.

**FIGURE 1:**
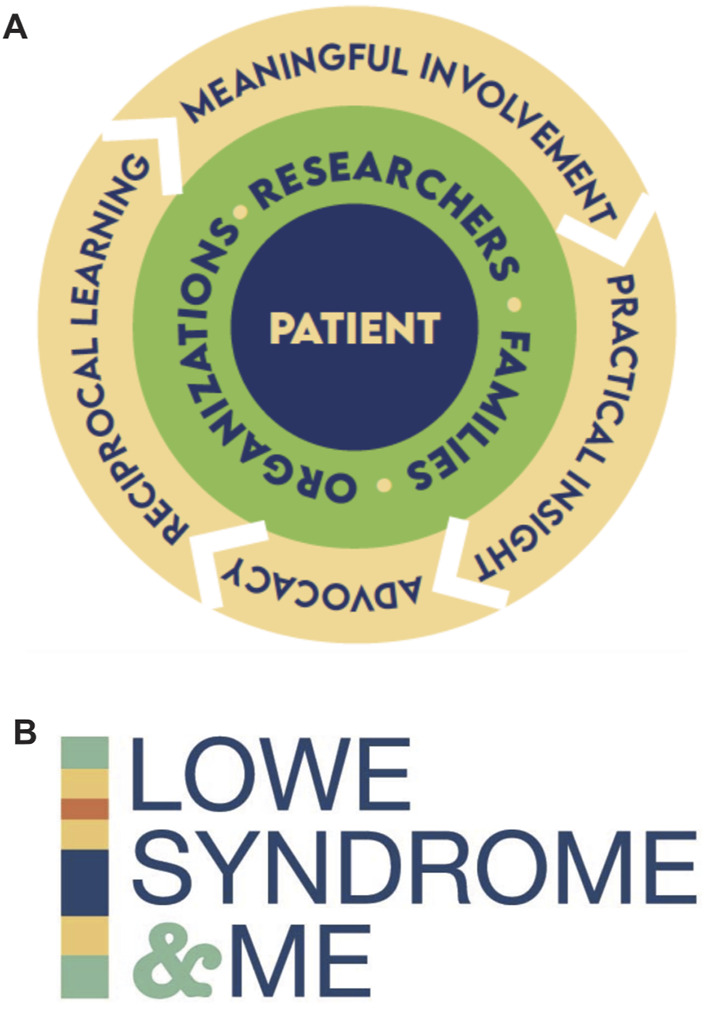
Rationale and graphics associated with the project **(A)** A cyclical process that centers on LS patients **(B)** Logo development featured a segmented line representing the chromosomal location of the defect in the OCRL1 gene. Graphics associated with the project based on the location of *OCRL* gene on chromosome X.

To open communication, deepen understanding, and connect as a cohesive group, participants from all groups decided to collaborate by co-creating a project to share their respective experiences of living with, advocating for, or studying LS (Figure 1A). A research team proposed a collaborative series of videos that center on the LS patient and capture an authentic narrative from each of the three key stakeholders (Figure 1A). This video series, titled Lowe Syndrome and Me, gathered researchers, affected families and supportive organizations to humanize each group and authentically share their stories. The video series, Lowe Syndrome and Me, is accessible online and used on social media. It aimed to develop new stakeholders’ networks, provide primary information about the condition, raise LS awareness and funding, and develop new skills for all participants. Branding included a logo featuring a segmented color bar that replicated the *OCRL 1* gene location on the X-chromosome (Xq26.1) where the gene mutation for Lowe syndrome exists (Figure 1B).

Patient and public involvement (PPI) refers to the active and meaningful involvement of patients, carers, their advocates, and the public in shaping, planning, designing, conducting, and disseminating research, ensuring their perspectives and experiences inform all research processes. PPI is a growing and impactful approach, most often used as a collaborative strategy in clinical research, recognizing those with lived experience as key partners, rather than entities within the research process. In this approach, research is done “with” rather than “for” those affected by the disease (Buchanan et al., 2022; Burke et al., 2023). The project described here centered on the co-production of a video series as an educational advocacy initiative and partnership between researchers, affected families and support organizations. It shares PPI clinical research objectives including support for meaningful involvement, practical insight, advocacy, reciprocal learning, and capacity building through an empowering partnership (Savas et al., 2022; Burke et al., 2023; Bevan et al., 2024; Thomson et al., 2024). There are also aspects of ensuring responsible research and innovation, where even early-stage research responds to the needs and lived experience of the communities it serves, as it can be unpredictable when research findings of direct impact can occur (Clements-Brod et al., 2022).

Those with lived experience have unique perspectives and valuable knowledge of daily needs and care that elevate support and family advocacy (Fagan et al., 2016; Buchanan et al., 2022; Savas et al., 2022; Bevan et al., 2024). The work of researchers can answer questions about the basis of disease and bring routes to new medications and therapies to ease symptomatic barriers and improve wellness, which provides hope for the future as well as practical benefits. The benefits of PPI initiatives include merged knowledge and support, open pathways for collective communication and shared understanding through meaningful dialog, and forged advocacy in a mutually beneficial way, all leading to better outcomes (Chudyk et al., 2018; Savas et al., 2022). It allows the visibility of family perspectives to inform researchers, a user-friendly approach to research relevance to inform families and creates advocacy opportunities for education and funding. This genuine partnership extends its reach through public engagement and outreach by spreading knowledge and experiences using patient-friendly dissemination channels (Fagan et al., 2016). Involvement with communities and families, in turn, can provide a sense of value, connection and motivation to researchers, whose work can seem removed from society due to its highly technical nature. Moreover, basic researchers are missing the experiences of clinician-scientists in patient care, where everyday life experience can provide a better representation of the patient experience compared to reading the clinical literature (Ketcher et al., 2021; Arumugam et al., 2023). Thus, direct interactions with patients and caregivers give researchers an additional source of understanding and motivation.

Initiatives involving PPI should include mindful selection to recruit family participants who capture a spectrum of experiences and needs (Fagan et al., 2016; Buchanan et al., 2022; Marcu et al., 2023). Diverse representation of family voices avoids a narrow perspective of needs and experiences for the entire group (Bevan et al., 2024; Thomson et al., 2024). Mechanisms need to support patient and researcher engagement strategies from a variety of stakeholder perspectives. The group dynamic should ideally be active, enthusiastic, weave together different expertise with an interest in common meaningful goals to engage the public, and advocate with shared decision making (Chudyk et al., 2018; Savas et al., 2022; Burke et al., 2023). All members need to feel empowered to effectively contribute (Thomson et al., 2024). Optimal PPI design for rare disease researchers and affected families utilizes online meetings allowing participants across a broad geographic range to strengthen diversity of backgrounds and experiences while eliminating geographical barriers, cost of travel, and childcare needs (Bevan et al., 2024). Making joint decisions with a smaller group design ensures full participation and involvement, balancing feasibility and accessibility (Thomson et al., 2024). A common facilitator ensures accuracy, consistency, dependability and accountability with shared guidance during the process (Marcu et al., 2023).

## Project methodology

### Participants

The parents’ patient group and advocacy group were recruited with the help of the Lowe Syndrome Association (LSA), United States. The participants were four mothers and one father, all with a son with Lowe syndrome. The affected boys ranged from 2 to 28 years old and included one toddler, one set of school-aged twins and two young men in their 20s. Locations of families included the northeastern region, midwest region and Hawaiian Islands (United States).

Five researchers from the Gurdon Institute (University of Cambridge, United Kingdom), including a group leader, committed to this project. The project was led and managed by the research institute’s public engagement team.

### Advisory group

The advisory group, composed of one adult patient, two family group members, and two researchers, was responsible for providing recommendations to the project participants and assessing the quality and ethics of the video series before dissemination on the internet and social media platforms.

### Public engagement team

**FIGURE 2:**
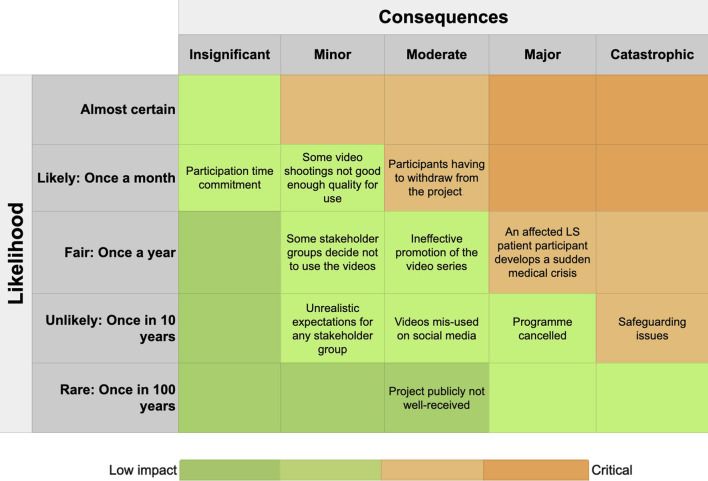
Risk assessment of running the project. Consequences and risks of harm from the project.

The project was led and managed by the research institute’s public engagement team who considered the balance of risks and benefits of implementing the project (Figure 2). They developed the project timeline and budget, recruiting participants, the video trainer, the video editor and the illustrator, liaising with all stakeholders, organizing the meetings and training sessions, assessing the video editing and script writing, getting feedback from the advisory group, disseminating the video series and evaluating the project.

### Meetings

Participants met online via the Zoom platform at a time chosen to best accommodate the patient and advocacy groups. They participated in a series of three one and half-hour meetings to identify and develop a project that could help them build bridges between the LS and scientific communities and generate a new tool to raise awareness about LS. The public engagement team facilitated all meetings to support the groups and moderate power dynamics.

### Project

The participants identified that videos created by each group showcasing their unique perspectives on LS and shared on social media and the internet could encourage interaction among the different groups. These videos would also provide essential information about the condition and help raise awareness and funding for LS. We aimed to give each group complete control over their production process. To accomplish this, all participants received training from a professional video specialist. They were tasked with filming their own videos and writing their own scripts, with support from the public engagement team. A video editor then compiled the materials provided by each group to create three videos ranging from 2 min and 30 s to 3 min and 30 s long, featuring a voiceover recorded by a member from each group. An additional short animation was co-designed by the groups and created by an illustrator to provide accurate and essential information about the LS condition to the LS community and the public.

### Dissemination

The video series was promoted on the Gurdon Institute’s Instagram, the patient group’s Facebook pages, and the participants’ social media accounts. It was also made available online on the Gurdon Institute’s YouTube channel. Additionally, the LSA, United States and the Association Française du Syndrome de Lowe (ASL) in France featured the animation on their websites. The project was highlighted at the Lowe Syndrome Research Symposiums 2023 and 2024, Cambridge Rare Disease Network RareSummit 2023 and RareFest 2024, in Masters taught courses at the University of Cambridge, at and the UK’s national public engagement conference, Engage 2024.

### Honorarium

The project adhered to the PPI National Institute for Health and Care Research United Kingdom standards, and its budget included financial compensation for patient group participants to acknowledge their time, involvement, and potential childcare expenses.

## Project impact

After completion of the project, a survey was emailed to participants in the Lowe Syndrome and Me video project to capture feedback on the process, participation, and perceived impact with qualitative questions added to deepen understanding. Of the six survey respondents, four were from the patient and family group, including one who is also the president of the national patient organization, and two were from the researcher group. The project’s overall rating revealed high satisfaction with a mean score of 95/100 with four out of six respondents submitting a score of 100.

Participants found significant value in their choice to participate, reflected in a mean score of 97/100. Motivation for participation included educating others about LS, bringing awareness to LS, helping those affected by the syndrome, forming a path to communicate the science, and creating an opportunity to take part in a great piece of work. When asked what each respondent got out of participation, they stated: “it helped to reflect on lived experience”, “it would be helpful to share when onboarding staff”, “it defined how to communicate clearly what science means to non-scientists”, and “included learning how to make research more accessible to the general public”. It also sparked remembrance for a parent of a young man with LS of how much had been overcome in the LS journey.

Participants found value in the process of making the videos. Learning how to best communicate science to the general public, the ability to use reflection for training others, and making research more accessible highlighted the impact of this video series as a learning and education tool. One parent of a young man with LS shared the emotional impact of memories when seeing videos of the younger boys. Training was well received, rating a mean score of 92/100 with four choosing a score of 100.

To measure impact, respondents were asked if their perception of LS or LS research changed after participation. This impact varied with a mean score of 50/100 with a remarkably broad range of scores from 5 to 100. Reciprocal learning was reflected in comments from each group. A researcher commented “Watching the other videos showed me a lot about the experience of living with/around LS” and a parent shared “My perception of LS has not changed since it is a daily occurrence, but the research aspect was interesting.” This highlights how participants deepened their overall understanding through this shared partnership. One parent noted that the educational impact extended beyond the participant group, reflecting that participation provided “an amazing way to share the information with friends, family, others who may not understand all it entails”. Value was also noted in the final series of videos with a high mean value rating of 95/100. Perceived value included the opportunity to share the information with others, inform family and friends, raise public awareness, and learn from the other partners through video series. It was also mentioned that the family and LS videos “humanized” the syndrome.

When asked what could be done differently, four of six responded that nothing could be changed. Overall, most participants hoped the video series would increase awareness of LS by boosting understanding, compassion, and hope, increasing funding, and raising a broader awareness of this condition and why more research is needed.

Through survey responses, evidence reveals that participants benefited from PPI through meaningful insight, purpose, and reciprocal learning (Bevan et al., 2024; Buchanan et al., 2022; Fagan et al., 2016; Savas et al., 2022). Collaborative partnerships and writing through online planning and creative meetings empowered participants to contribute in a meaningful way. Deadlines were met, and project goals were achieved with enthusiasm and purpose. The desire to help other families was reflected in the survey and mentioned in the meetings. This is not uncommon with LS families often geographically living in isolation. Participating researchers conveyed increased motivation in their research, new recognition of societal benefit of their endeavours and an appreciation of the importance of inclusion in their work. The video dissemination also prompted questions from researchers working in unrelated areas about the severity of the condition and whether any voluntary efforts from them could contribute to elucidating disease mechanisms or identifying a meaningful treatment.

The video was welcomed by the French association and presented during the general assembly and at conferences. The animation video was also dubbed in French and adopted by the French patient association for their website, showing the project’s wider value beyond the immediate participants.

## Discussion

Those with lived experience have unique perspectives and valuable knowledge of daily needs and care that elevate support and family advocacy (Fagan et al., 2016; Buchanan et al., 2022; Savas et al., 2022; Bevan et al., 2024). The dedicated work of researchers can bring mechanistic insights, new medications and therapies and hope for the future. The benefits of PPI initiatives include merged knowledge and support, open pathways for collective communication and shared understanding through meaningful dialog, and forged advocacy in a mutually beneficial way, all leading to better outcomes (Chudyk et al., 2018; Savas et al., 2022). It allows the visibility of family perspectives to inform researchers, a user-friendly approach to research relevance to educate families and creates advocacy opportunities for education and funding. This genuine partnership extends its reach through public engagement and outreach by spreading knowledge and experiences using patient-friendly dissemination channels (Fagan et al., 2016).

The role of PPI projects is to identify and share perspectives and broad issues of importance by participant groups, build communication and trust for each other and the process, and create inclusive ways of working together (Savas et al., 2022; Burke et al., 2023; Bevan et al., 2024). Active and meaningful collaboration allows priority setting for knowledge transition and advocacy through authentic meaningful involvement with a clear purpose (Chudyk et al., 2018; Burke et al., 2023). Extending beyond the partnership initiative for rare diseases, the family and patient involvement can reduce the sense of isolation and loneliness caused by the diagnosis of an ultra-rare syndrome (Marcu et al., 2023). The participating families’ mutual desire to help other affected families is strong (Savas et al., 2022).

However, PPI projects face barriers such as time, training, financial constraints, or lack of stakeholder interactions. For example, families affected by LS are often geographically isolated with a team of doctors who are seeing a LS patient for the first time. With this rare occurrence, the interest in research is a small and dispersed yet robust and active research community. Within the study, locations of researchers in Cambridge, United Kingdom and participating families spread across the United States required online communication and training. Only a limited number of participants could reasonably join the project, so the range of ability was confined to the group of individual LS patients selected. Medical complications with LS are often unpredictable and limit availability.

The highly technical nature of research limits the extent of common language (Clements-Brod et al., 2022; Marcu et al., 2023), and a related issue is how early is too early for patient involvement in research. Engaging too early risks being complex and not relevant, yet the absence of it can lead to backlash or alienation and early engagement helps trust develop (Clements-Brod et al., 2022; Burke et al., 2023). A dialogue built over a period of time can build relationships and mean that a common understanding can be developed. One observation in our video series was the novelty of the laboratory equipment and environment from lay participants. Familiarity with the research process can build trust and lead to donations for research.

Diverse representation of family voices avoids a narrow perspective of needs and experiences for the entire group (Bevan et al., 2024; Thomson et al., 2024). Initiatives involving PPI should include mindful selection to recruit family participants who capture a spectrum of experiences and needs (Fagan et al., 2016; Buchanan et al., 2022; Marcu et al., 2023). Mechanisms need to support patient and researcher engagement strategies from a variety of stakeholder perspectives. All members need to feel empowered to effectively contribute (Thomson et al., 2024). Optimal PPI design for rare disease researchers and affected families utilizes online meetings allowing participants across a broad geographic range to strengthen diversity of backgrounds and experiences while eliminating geographical barriers, cost of travel, and child care needs (Bevan et al., 2024). Making joint decisions with a smaller group design ensures full participation and involvement, balancing feasibility and accessibility (Thomson et al., 2024).

A successfully executed video series was created that shared perspectives between LS patients, caregivers, advocates, researchers and animation professionals. Holding all meetings virtually opened opportunities for participation across the United States and the United Kingdom, telling a diverse LS story (Bevan et al., 2024). A common facilitator ensured accuracy, consistency, dependability and accountability with shared guidance during the process (Marcu et al., 2023). Using a common facilitator also streamlined the process so participants could focus on collaborative writing and creation and directional clarity, with a partnership emphasis designed to promote advocacy (Chudyk et al., 2018; Savas et al., 2022). The role of the facilitators accommodated many schedules across a span of time zones from Hawaii-Aleutian Standard Time to Greenwich Mean Time, setting priorities, and keeping participants accountable. Reciprocal learning was noted in survey responses, and the use of patient-friendly distribution expanded the reach.

As research advances, the need for key PPI partnerships expands. There will continually be new stories to tell surrounding this ultra-rare syndrome. Opportunities include expanding the family experience through the addition of more families, adding the direct voices of some of the young men living with LS, and showcasing new research progress to pharmaceutical companies and clinical stakeholders. New distribution avenues should also be explored. This project model could be replicated for any partnership of patients, caregivers, researchers and advocacy support organizations that have a common goal and strong desire for shared partnership. One hope is that the partnership has the capacity to expand reach by enticing new researchers into the field, giving hope to families and providing both researchers and families with the information and relationships to discover additional ways to extend wellness.

## Data Availability

Publicly available datasets were analyzed in this study. This data can be found here: https://www.gurdon.cam.ac.uk/public-engagement/lowe-syndrome-me/.
